# “Antidepressants and movement disorders: a case of an adolescent with major depressive disorder”

**DOI:** 10.1192/j.eurpsy.2025.1161

**Published:** 2025-08-26

**Authors:** M. Ríos Vaquero, M. Esperesate Pajares, O. Martin Santiago, G. Lorenzo Chapatte, L. Rojas Vázquez, A. Monllor Lazarraga, L. Sobrino Conde, B. Rodriguez Rodriguez, N. Navarro Barriga, M. Fernández Lozano, M. J. Mateos Sexmero, P. Pando Fernández, M. Calvo Valcarcel, M. Andreo Vidal, P. Martínez Gimeno, L. Del Canto Martinez, F. J. González Zapatero, M. D. L. Á. Guillén Soto, A. Aparicio Parras

**Affiliations:** 1 Psychiatry, Hospital Clínico Universitario de Valladolid, Valladolid, Spain

## Abstract

**Introduction:**

Drug-induced movement disorders are a rare side effect of which some cases have been reported and published. It may be due to antidepressants, antipsychotics or other drugs. In these cases it is important to realize differential diagnosis, identify the medication that may have caused it and implement appropriate management to solve it.

**Objectives:**

To present the case of a 16-year-old adolescent diagnosed with major depressive disorder with movement disorders after treatment with escitalopram.

**Methods:**

A literature review was conducted on movement disorders and treatment with antidepressants in child-adolescent population.

**Results:**

Our case is about a 16-year-old adolescent who is living with his family and he is studying at the high school. The patient has no personal psychiatric history and in his family only his father has an adaptive disorder treatment. The first time he comes to consultation, he said that in the last few weeks he has anxiety attacks in different environments and an increase in basal anxiety. He has been in a low mood for about two years with apathy, which has increased in recent weeks. He is diagnosed of major depressive disorder and we decided to continue with escitalopram 10 mg and chlorazepate dipotassium prescribed a week ago by his family doctor. After one month, he goes to the second consultation with difficulty in walking, stiffness and instability that is related to the introduction of treatment. In addition, he comments on mild improvement of anxiety and mood. Due to the timing of the onset of movement disorder and the onset of escitalopram, it is decided to suspend it. Chlorazepam dipotassium is maintained and short-term for evaluation of evolution. In the following consultation, no movement disorder symptoms are seen and the onset of another antidepressant with a different course of action is assessed.

**Conclusions:**

Drug-induced movement disorders as in the case described are a rare side effect of which some cases have been reported and published. Different studies reveal the different behavior of antidepressants in the adult and child-adolescent population. In a meta-analysis on antidepressants and depression in the child-and-youth population (1), the results indicated that sertraline, escitalopram, duloxetine and fluoxetine could be considered as the first option although cases with associated extrapyramidal symptoms (EPS) were reported. According to a study on the association of antipsychotics, antidepressants with movement disorders in children and adolescents (2), the risk-benefit profile of antipsychotic use should be considered as an adjuvant to reduce EPS. In addition, in a post-marketing study (3), a potentially harmful association was found between movement disorders and the use of antidepressants mirtazapine, vortioxetine, fluvoxamine, citalopram, paroxetine, duloxetine, escitalopram, fluoxetine, sertraline, venlafaxine, among others.

**Disclosure of Interest:**

None Declared

